# Insights into Population Health Management Through Disease Diagnoses Networks

**DOI:** 10.1038/srep30465

**Published:** 2016-07-27

**Authors:** Keith Feldman, Gregor Stiglic, Dipanwita Dasgupta, Mark Kricheff, Zoran Obradovic, Nitesh V. Chawla

**Affiliations:** 1 University of Notre Dame, Computer Science and Engineering, Notre Dame, 46556, USA; 2 University of Notre Dame, Interdisciplinary Center for Network Science and Applications (iCeNSA), Notre Dame, 46556, USA; 3 University of Maribor, Health Sciences, Maribor, 2000, Slovenia; 4 University of Maribor, Electrical Engineering and Computer Science, Maribor, 2000, Slovenia; 5 St. Joseph’s Regional Medical Center, Mishawaka, 46544, USA; 6 Temple University, Data Analytics and Biomedical Informatics Center, Philadelphia, 19122, USA; 7 Wroclaw University of Technology, Wrocław, 50-370, Poland

## Abstract

The increasing availability of electronic health care records has provided remarkable progress in the field of population health. In particular the identification of disease risk factors has flourished under the surge of available data. Researchers can now access patient data across a broad range of demographics and geographic locations. Utilizing this *Big* healthcare data researchers have been able to empirically identify specific high-risk conditions found within differing populations. However to date the majority of studies approached the issue from the top down, focusing on the prevalence of specific diseases within a population. Through our work we demonstrate the power of addressing this issue bottom-up by identifying specifically which diseases are higher-risk for a specific population. In this work we demonstrate that network-based analysis can present a foundation to identify pairs of diagnoses that differentiate across population segments. We provide a case study highlighting differences between high and low income individuals in the United States. This work is particularly valuable when addressing population health management within resource-constrained environments such as community health programs where it can be used to provide insight and resource planning into targeted care for the population served.

Today we are witnessing a shift in the landscape of modern healthcare. The rapid emergence and adoption of Electronic Medical Records (EMR) has led to a sundry of analytic technologies. These technologies utilize aggregated EMR’s from numerous individuals in conjunction with machine learning and statistical techniques to provide personalized diagnoses based on a patient’s specific health conditions, clinical decision support systems, and numerous other tools employing secondary uses of EMR data^1^,^2^,^3^,^4^,^5^.

While these methods and technologies have provided advancements to both diagnostic accuracy and patient safety, the research thus far has been limited in leveraging data-driven methods to understand population level health dynamics. The EMR data, indeed, provides an exciting opportunity to gain a more complete and holistic understanding of a population segment. This not only offers an understanding of the risks faced by the population at large, but also offer insights into more effective resource management and application.

Traditionally, those studies that do attempt to address the issue at a population level have done so with respect to specific diseases such as diabetes, cardiovascular disease and mental disorders^6^,^7^,^8^. Further these population studies often evaluate attributes outside of an individual’s direct control, such as age, gender, and ethnicity. However, prior population health works have shown these attributes account for only a small portion of an individual’s overall health condition. In fact, the set of factors contributing most notably to an individual’s health have been linked to their socioeconomic “circle,” or as stated by Dahlgren and Whitehead, the set of material and social conditions in which people live and work^9^.

One factor that has drawn a great deal of attention from population health researchers, is that of income. Two fundamental studies related to population income disparity were performed by Adler and Marmont respectively^10^,^11^. In their work Marmot *et al*. go so far as to claim that “all modern analysis must now ‘control for’ social class as they do for sex”. Since the publication of these works, there have been a number of additional studies investigating the relation of income to health more closely^12^,^13^,^14^. While these studies have provided a detailed evaluation into how socioeconomics can influence a population’s health, they fail to address the major factor of identifying specifically *which* diseases the population is at risk for.

*The methodology presented in this work addresses this exact deficiency*, *helping to answer the question*: what are the distribution of diseases, including comorbidities, of varying subgroups within a population? An understanding of a population’s high-risk diagnoses has been shown to offer substantial benefits at both an administrative and community level. From an administrative perspective, population health can have a direct impact on public policy. A study by Tarlov proposed four frameworks that must be used in conjunction to provide guidance for constructing effective public policy, one of which is constructed from the “determinants of population health”^15^. Building on ideas similar to Dahlgren, Tarlov believes social/societal factors account for more than half of all influences on a population’s health. He adds to the importance of this understanding by posing that population interventions are unlikely to be successful without social/societal change.

On the community side, diagnosis specific inference can provide a direct impact on individual care. Community healthcare operations often struggle with restricted healthcare budgets or limited resource access, and as a result the allocation of their current resources has become increasingly important. An early study by Birch *et al*. detailed a need-based healthcare resource allocation system, which like many early works focused on population attributes such as age and sex^16^. With their work Birch *et al*. demonstrated that external data could be utilized to improve healthcare allocation, and offered that the next challenge was to “construct indicators of relative need” for a population. Building from this, we believe the ability to identify a population’s specific high-risk diagnoses could provide more effective resource allocation and treatment plans.

Although the quantity and quality of healthcare data is increasing, the analysis needed to obtain the diagnosis specific insights still present some challenges. Just as the complexities of human physiology and the unique nature of individual’s medical history have provided difficulties in the field of personalized medicine, the wide range of ethnicities, genders and ages between individuals offers a similar obstacle to the field of population healthcare. Variations such as these become particularly evident as we analyze populations stratified on attributes such as income, which fall on the outer edge of an individual’s socioeconomic circle.

In an attempt to address these complexities many studies have drawn on the field of network science. One of the first approaches, a study by Hidalgo *et al*., constructed networks based on disease comorbidities^17^. In these early works Hidalgo *et al*. and others demonstrated how diagnosis networks could be used to quantitatively study the properties of comorbid diseases^18^,^19^,^20^,^21^. Over the past decade network-based techniques have continued to evolve, with applications ranging from the identification of cancer-specific disease comorbidities, to the utilization of diagnostic data to identify the skeletal components that contribute to pathological disequilibrium in dental patients^22^,^23^,^24^,^25^.

Beyond the macro-level physical interactions found in diagnosis networks, extensive work has also been performed on utilizing networks to model the complex interactions that occur on a molecular-level^26^,^27^,^28^,^29^. Further, in addition to their diagnostic applications, exciting new work has emerged with a focus on utilizing networks to model human physiology. These networks aim to inform our knowledge of the interdependency among several organs systems, and their function within the complex biological systems of the human body^30^,^31^,^32^.

It has become increasingly clear that population health management can no longer operate under a one-size-fits-all paradigm. In this work we leverage the network based framework, which allows for the analysis of the deviations in comorbidities and specific diagnoses between population subgroups. We introduce a network-focused metric termed fold-change. The fold-change provides a normalized metric of the over-representation for diagnosis comorbidity pairs between two generalized population subgroups. Through the analyses presented we will establish not only the utility of the fold-change metric in identifying high-risk diagnoses targeted for specific population subgroups, but also demonstrate how partitioning the network using the fold-change metric as edge weights can help to uncover new and interesting diagnosis patterns not discernible through naive partitioning techniques. We provide a case study of patients from high and low income populations, based on median household income, within the United States. Additionally, we provide an extended analysis detailing the effects of further stratification based on insurance of the patient, discovering potentially cost saving disease hubs that could be used to guide the preventive actions of healthcare organizations and policy makers.

## Results

The evaluation of the network-based technique proposed in this work was broken down into two distinct analysis, each of which investigated a different aspect of the diagnosis variations between the population subgroups. These subgroups represent individuals in the highest and lowest quartiles of median income across the United States. A summary of each analysis and the corresponding results can be found in the respective sections below.

The foundation for each of the following analyses was the creation of a diagnosis network to help standardize a disease intearaction representation as also done in the related works. As such, prior to detailing the analysis results we offer a brief overview of the experimental framework, providing context around the specific attributes of the networks utilized throughout the work.

### Network Construction

In the simplest form a network is a relational data representation. This representation is comprised of a set of entities, known as nodes, and connections between pairs of nodes, known as edges. Within the context of the diagnosis networks used in this work, each node represents a unique diagnosis code (provided in the ICD9-CM international standard), and co-morbid diagnoses share an edge. Diagnoses are comorbid if they co-occur in a patient.

Additionally, the edges of the network were assigned a weight corresponding to the frequency at which the diagnosis nodes occurred comorbidly across all patients of the particular subgroup. It should be noted the edge weights of each diagnosis pair were normalized, the complete formalization of this process can be found in the *Study Data and Methods section*.

### Comorbidity Analysis

Our first analysis focused on the identification of over-represented network edges (comorbid diagnoses) for a particular population subgroup. The over-representation of a specific edge was quantified by a normalized ratio of the corresponding edge weights in each of the two subgroup networks, a quantity henceforth referred to as “fold change”. A detailed discussion of the fold change metric can be found in the *Study Data and Methods section*.

**Figure 1:**
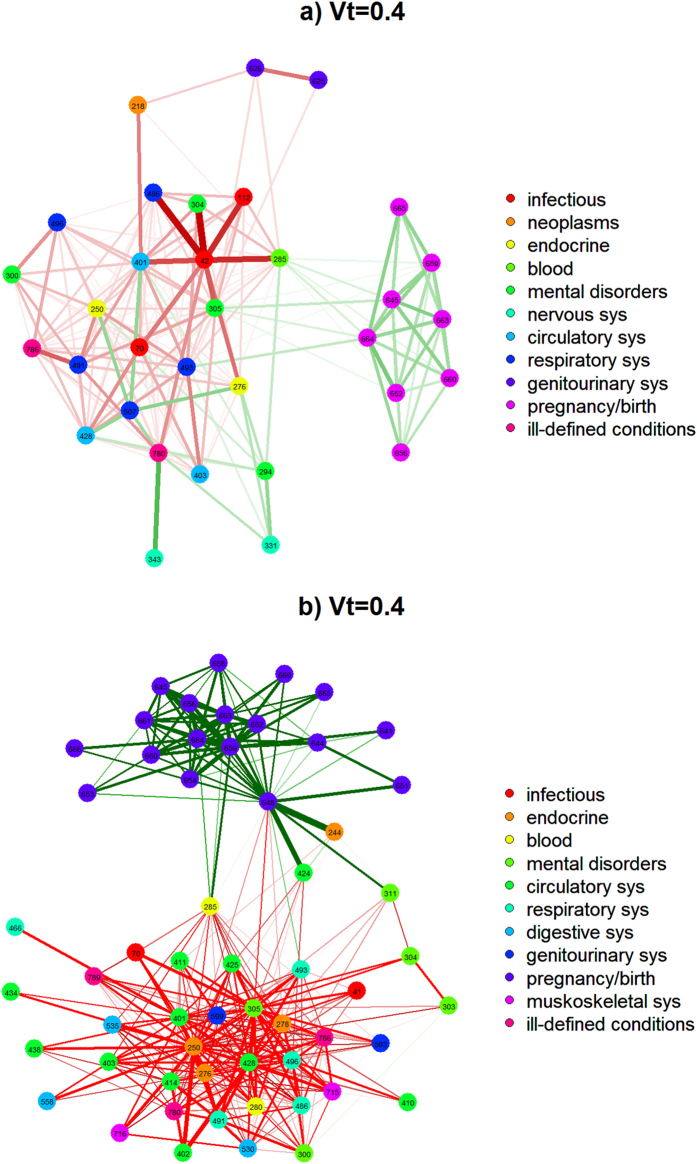
Presents a visualization detailing the diagnosis fold-change between the low and high income populations in a single network representation. (**a**) Complete Network Visualization comparing low income and high income populations. (**b**) Network Visualization without pregnancy/birth related diagnoses.

Figure 1a presents a single-network representation of the diagnosis fold-change between the low and high income populations. The numbers within each node represent the respective ICD9-CM diagnosis code, and the edge coloring conveys whether the comorbid diagnoses were over-represented in the high or low income subgroup. Edges where the fold-change ratio was greater than 1 for the high income subgroup were colored green, and those where fold-change ratio was greater than 1 for the low income subgroup were colored red. For clarity a fold-change value over 1 represents the case where the edge weight, representing the normalized comorbidity frequency between two diagnoses, is greater in a particular subgroup.

One benefit of the network visualization seen in Fig. 1a stems from the ability to quickly identify specific groupings of diagnoses. The figure was generated using what is known as a force-directed layout, where nodes are positioned in an effort to separate groups of adjacent (connected) nodes in a visually interpretable manner. As a result the prevalence of complications for pregnancy and childbirth in a group of high income female patients could be seen with relative ease. To account this an additional visualization is provided in Fig. 1b, which excludes pregnancy/birth related diagnoses, allowing a far more detailed view of the network composition.

A review of the low income population’s over-represented edges revealed a high prevalence of mental disorders (*schizophrenia*), chronic diseases (*obesity*, *diabetes*) and drug related abuse and dependence. Conversely over-represented edges within the high income population were comprised almost entirely of pregnancy related conditions, ranging from labor conditions to delivery trauma. Conditions including *obstructed labor* represent a risk to the mother while others such as *umbilical cord complications*, and *malposition* indicate risk to the fetus.

For reference we have provided the top 10 diagnosis code pairs ranked by highest fold-change in Tables 1 and 2 for the low and high populations respectively. It is important to note that all edges listed in the top 10, as well as all edges shown in Fig. 1, are significantly over-represented at p < 0.001.

### Diagnosis Analysis

Next we extend the comorbidity results to investigate the specific diagnosis nodes which comprise the over-represented edges of each subgroup.

Focusing on the low income population’s top 10 over-represented edge pairs (Table 1) we find multiple occurrences of nodes that constitute drug abuse or dependence. Analyzing the top 10 over-represented edge pairs for the high income population (Table 2) reveals multiple occurrences of edges containing the specific ICD9-CM code 664: *Trauma to perineum and vulva during delivery*. Trauma code 664 contained edges to a number of conditions which included *known or suspected fetal and placental problems*, *abnormality of forces of labor*, and *umbilical cord complications*.

### Network Metric Analyses

Thus far, the analyses presented in this work have demonstrated the utility of the proposed fold-change metric. These analyses have highlighted how fold-change rankings can be utilized to identify significantly over-represented diagnoses, which can be beneficial in differentiating the health concerns of various population subgroups. However, with our final analysis we take the fold-change metric one step further, demonstrating that combined with a standard network analysis (betweenness-centrality), it can be used to discern core-differences between the population groups. These differences are not immediately evident on the network not weighted by fold-change metric. Betweenness-centrality is a network metric which provides a normalized measure of the global importance of a node in communicating between pairs of nodes in the network, considering the shortest paths^33^.

Utilizing the methodology detailed in the Network Construction section prior, two networks were constructed representing the high and low income populations. These networks were created utilizing a naive partitioning, in which the over-represented comorbidity pairs of patients in the high income population were added as edges to one network, while the over-represented comorbidity pairs of the low income population were added as edges to the other. The standard metric of betweenness-centrality was calculated on each network independently, and the top 10 diagnosis nodes ranked in decreasing order by betweenness-centrality are presented within Table 3. As can be seen from the results, there are a number of high-ranking diagnoses which coincide with the over-represented diagnoses identified by the fold-change rankings. For the high income population these included pregnancy related conditions such as *Other current conditions in the mother classified elsewhere*, while *Nondependent abuse of drugs* and a number of chronic conditions receive high centrality scores within the low income population.

Looking to Tables 4 and 5, we again provide rankings of betweenness-centrality calculated on both the low and high income population networks. However, in this case, each network underwent a range of pruning thresholds, at which all edges with a fold-change below the specified threshold value were removed. A complete overview of the process can be found in the Methods section. As a note, the high income population (Table 5) threshold at 2.0 has only two non-zero centrality scores after pruning, the remaining three spots are intentionally blank. It is overtly evident that the threshold value has a profound impact on the network structure, as the betweenness-centrality rankings undergo significant reordering across the range of thresholds. This can be seen in the high income population where diagnosis such as *diabetes mellitus* drops from Rank 1 to 5, while *schizophrenic disorders*, which was previously ranked outside the top-5, rises to rank 2. This phenomena is mirrored in the low income population where diagnoses such as *hypertension* and *cardiac dysrhythmias* were initially ranked 1 and 2, quickly fall outside the top 5, and are replaced by pregnancy related conditions such as *trauma to perineum and vulva during delivery* at higher threshold values. A deeper discussion as to the implications of these changes can be found in the Discussion section.

Finally, in an effort to compare the observations from our fold-change ranking, with those detected using network structure alone, we performed community detection on the complete high and low income population networks. The results of these analyses can be found in the supplementary material.

## Discussion

A review of the relevant literature reveals strong support for the observations derived from the income-stratified networks above. Beginning with the low income population, prior work has linked individuals’ with lower socioeconomic status to a higher prevalence of chronic disease such as diabetes and obesity^34^,^35^,^36^. A study by Bassuk *et al*., which focused on the “Prevalence of Mental Health and Substance Use Disorders Among Homeless and Low-Income Housed Mothers”, found a significant increase in the rate of both mental health and substance use disorders amongst the low-income population when compared the general population. Additionally, the study highlighted an increased prevalence of the mental disorder schizophrenia amongst homeless solitary women^37^.

Likewise we find support in the literature for the over-represented diagnoses of the high income population. As noted prior, pregnancy related conditions constituted almost the entirely of the high income populations over-represented conditions. We conjecture the preponderance of pregnancy related conditions may be attributed to increased access to prenatal care, as many of the identified conditions were likely diagnosed during an expectant mothers prenatal visits over the course her pregnancy. It is well established that due to the cost of these visits, and limited access to resources, lower socioeconomic populations often times fail to receive prenatal care at the appropriate times, if at all^38^.

In a similar fashion we find that a survey of diagnosis nodes corresponding to these over-represented edges can be supported by medical literature. The low income population’s prevalence of drug abuse or dependence nodes are connected to well documented comorbid conditions such as hepatitis and recurrent seizures^39^,^40^,^41^. Additionally a closer investigation of the diagnosis pairs yields another less apparent condition, kidney disease. Recent work has begun linking the long established toxic effects of drug use and abuse to the development of chronic kidney disease, fostering additional support for the power of our network-based technique to identify clinically useful comorbidities within a population subgroup^42^,^43^,^44^. For the high income population we evaluated conditions that occurred comorbidly with the diagnosis *Trauma to perineum and vulva during delivery* (ICD9-CM code 664), which appeared multiple times in the set of over-represented edges. Investigation revealed that women who experience the pregnancy complications comorbid to code 664 have a significantly higher probability of their child being delivered with the aid of forceps^45^. This is particularly noteworthy as prior work has associated delivery with forceps with a 10-fold increased risk of perineal injury^46^. These findings again highlight the power of our network technique to identify high-risk diagnoses for a population subgroup, in this case representing high income pregnant women.

Moving to the network-focused analyses, as the overarching goal of this work was to provide a technique through which differentiating and representative diagnoses between two population sub-groups could be identified, the measure of betweenness-centrality is an excellent evaluation lens. In the context of co-morbidity network structures betweenness-centrality can be viewed as a measure of a diagnosis’s connectivity, i.e. the number of edge pairs which contain the diagnosis. Looking back over the betweenness-centrality results, one of the principle observations can be found in the stark difference between the centrality rankings on the unmodified and pruned networks for each population subgroup.

This differentiation is critically important as it demonstrates the utility of the fold-change metric in uncovering the core differences between population subgroups. By punning the network using the fold-change metric, we are able to remove peripheral edges for comorbidity pairs that have only a slight over-representation in either population. Recalculating the betweenness-centrality on these pruned networks offers us the opportunity to identify key nodes of a network representing highly polarizing comorbidities between the population subgroups.

Breaking down the results we can find explicit examples of where the identification of specific diagnoses would not have been apparent though analysis of the networks alone, but required the introduction of the fold-change metic into the analysis pipeline. For the high income population we see a ranking of 1, 2 and 3 for *diabetes mellitus*, *essential hypertension* and *nondependent abuse of drugs* respectively on the unmodified network. As noted prior these are certainly conditions which are to expected to be over-represented in a lower income population. However, these are also extremely prevalent diagnoses, particularly hypertension which can occur comorbidly with a multitude of diagnoses. Increasing the threshold value to 2, provides a number of interesting changes. First, prevalent diagnoses such as *diabetes mellitus* and *essential hypertension* fall in the rankings, indicating that the majority of their connections were only weakly over-represented between the two subgroups. While diagnoses such as *nondependent abuse of drugs* and *schizophrenic disorders* claim the top 2 spots. While less prevalent these diagnosis have a much stronger over-representation between the population subgroups, potentially helping to highlight avenues of care or resource distribution.

We find similar patterns upon analysis of the high income population’s results. While the unpruned network presents various cardiac conditions such as *essential hypertension*, *dysrhythmias* and *unspecified anemias*. Increasing the threshold highlights previously discussed pregnancy related conditions. At 1.5 we find *current conditions in the mother classified elsewhere*, and 2.0 presents *trauma to perineum and vulva during delivery*.

For completeness we ran two standard community detection algorithms WalkTrap and label-propagation. These analyses can aid in an understanding of how the underlying network structure may effect network metrics such as centrality measures, as it will help provide insights into the connectivity between various comorbidity patterns. As can be seen from the results (found in the supplementary material), community detection on the partitioned subgroup networks offers little insight into the populations diagnoses distribution. The majority of diagnoses are grouped into a single community, with only a select few placed into meaningful communities. Although this subset of communities may appear to highlight some similar occurrence patterns to the fold-change rankings, such as pregnancy related conditions, these occurrences are in fact an artifact of the highly interconnected subgraph of co-occurring diagnoses during pregnancy. It is clear that standard community detection alone fails to provide the ability to identify specific differentiations between population sub-groups, again highlighting the utility of the incorporating the fold-change metric into the analysis pipeline.

Through this work we have demonstrated and validated that the proposed network technique provides a clear differentiation in frequent diagnosis between population subgroups, in particular individuals in the upper and lower quartiles of income within the United States adult population. While this case study was focused on the identification of specific population health conditions based on varying socioeconomic levels, the network approach used for this identification can be easily extended to a multitude of population subgroups and external factors. As an example a similar analysis is provided in the supplementary material with patients further stratified by various insurance providers. It is important to note the scale of the data and observations discussed. Both the data used for this analysis as well as many of the cited works were collected at the national level. This was done intentionally in an effort to validate the observations and results of our technique against established medical literature acting as a ground truth. While national data is imperative for effective analysis of the general population, we believe this technique can be applied at the community level to start making differences, for example, to Accountable Care Organizations grappling for answers around resource allocation and budgeting. Our methodology may aid in identifying potentially unique comorbidities within a specific subgroup or population, allowing physicians and nurses to provide more accurate and targeted care for individuals they see on a daily basis as well as direct potential resources and information.

Finally, it is clear that this work is only a stepping stone to greater population heath management. For this type of analytics to truly take hold we will need the collaboration of data scientists, medical professionals and policy makers alike to produce, analyze and act on the insights gained through tools such as this. Looking forward as personalized treatment options have been key to improving the quality of patient care, a deeper understanding of the population health conditions will be critical for improving treatment options and resource management for a population.

## Study Data and Methods

### Data

For the analyses performed in this work we utilized the Nationwide Inpatient Sample (NIS) provided by the Healthcare Cost and Utilization Project (HCUP)^47^. At the time of this publication the NIS is the largest publicly available all-payer inpatient health care database in the United States. The NIS contains hospital discharge records for a stratified sample of patients from approximately 20% of United States hospitals, representing 44 different states (at the time of this publication) and has been cited in over two thousand different peer-reviewed journal articles.

Each record contains both the personal characteristics of the patient, including their age, gender, and race, and administrative information including their length of stay, and discharge status. Additionally, the NIS provides diagnostic information, including up to 15 (2003–2008 datasets) or 25 (2009) diagnoses, surgical and nonsurgical procedures. Diagnoses were coded using The International Classification of Diseases, 9^th^ Revision, Clinical Modification (ICD-9-CM)^48^. The ICD-9-CM coding methodology uses a taxonomy of five-digit codes, where the first three digits represent the general diagnosis and the remaining two digits can be used as modifiers describing factors such as location or severity. For this work we “collapsed” the codes, using only the first three digit general diagnosis. This allows us to both prevent similar diagnoses from being represented as multiple edges within the network, as well improve the overall interpretability of the results.

To construct the diagnosis networks used in our analyses we utilized a total of 21,662,600 patient hospitalization records representing the set of 9,306,956 high income and 12,355,644 low income records; the details of this partitioning from the HCUP data can be found in the Methods section below. These networks represent the co-occurrences of 934 unique ICD9-CM diagnosis codes between patients in the low and high income populations. Additionally it should be noted that for this analysis we considered records from only those patients with an age over 20 to exclude child related diagnoses and focus on the adult population.

## Methods

Before detailing the methodology used in the network construction and analysis it is important to detail the population being evaluated. As stated earlier this case study focuses on the comparison of low and high income individuals. The NIS income data is based on the median household income for a patient’s specific zip code. Under this definition an income level of 1 represents a reported income in the lowest quartile, while a 4 represents the highest median quartile at the individual’s respective location. The low income population subgroup was determined as the set of all patient records in the lowest median income quartile (1), while the high income population subgroup represents those in highest median income quartile (4).

To build the diagnosis networks, first we extracted the unique set of ICD9-CM comorbid diagnosis codes between patients in the low and high income populations. To account for the difference in population size the network edge weights were normalized using Equation 1. Here N represents the total number of records in a population (low or high income), while *C*_*ij*_ represents the number of co-occurrences of diagnosis *i* and *j* within the patients of each population respectively.


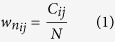


Next the ratios between 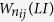 and 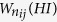 were computed to identify those diagnoses which occur in greater proportion amongst each of the populations. We define the ratio of diagnoses *i* and *j* between the two population subgroups as the *fold-change* (Equation 2). For clarity, the edge analysis performed in this work focuses on differences in diagnoses occurrence between each population, rather than the population on the whole. This is done in an effort to demonstrate the potential benefit of utilizing population level health records for specific community analysis. It should be noted that all normalized edge weights below the threshold 0.0001 were set to 0 to prevent high fold-change ratios from an extremely rare diagnosis.


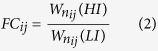


To aid in interpretation of the differences in the observed networks, the edge weights were normalized further to fall within the interval [−1, 1] using the transformation found in Equation 3. *W*_*HL*_ represents the edge weight calculated as a quotient of the high income population’s edge weight by the low income population’s respective edge weight and *W*_*LH*_ represents the opposite quotient. Finally, due to the density of the network to avoid over-saturation of nodes and edges and allow for an optimal visualization we introduce a visualization threshold *V*_*t*_. The threshold displays only those edges where *V*_*t*_ < *abs*(*W*_*Diff*_). It should be noted that while the threshold *V*_*t*_ was set experimentally to reduce the number of edges and related nodes with low weights for the visualizations provided, the analyses in this work were performed on the complete network.


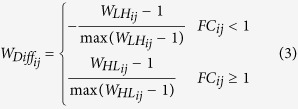


In order to determine statistical significance of the fold change results we performed permutation tests using the Fisher-Yeats shuffle algorithm^49^. For each diagnosis code pair we performed a 10,000 iteration shuffle. At each iteration we randomly shuffled the high and low income level labels at each occurrence and calculated the resulting *fold-change*. The resulting 10,000 fold-changes are averaged and compared to the true fold-change to obtain a Z-score evaluated at 95% significance level.

For our final set of analysis we perform a set of network analyses, including betweenness-centrality and community detection. Betweenness-centrality is a network metric, which on per node basis provides the proportion of shortest-paths through the network which pass through each node^50^. The mathematical definition is provided in Equation 4, where *SP*_*st*_ is the total number of shortest paths from node s to node t and *SP*_*st*_(*v*) is the number of those paths that pass through v.


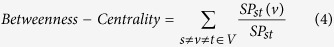


We began the betweenness-centrality analysis by computing node rankings on the complete network for both populations independently. The networks are undirected, but have edge weights corresponding to the fold-change metric detailed prior. For the pruning analysis the complete network was created for each population, and all edges below the specified fold-change threshold were removed. The betweenness-centrality was calculated at every step from 1–2 at intervals of 0.25.

It should be noted that all fold-change values are above 1. As noted fold-change is a normalized metric, indicating the level of over-representation of specific diagnosis co-occurance pattern between the population subgroups. As such, under represented comorbidities in one network (fold-change values less than 1) are presented in the second network where the reciprocal fold-change is utilized.

For our community detection we chose two widely utilized algorithms in Walktrap and label-propagation, both run with default parameters^51^,^52^. These algorithms represent two distinctly different approaches to discovering communities from the underlying network structure in an unsupervised manner, and thus can offer a fairly comprehensive overview of the types of diagnosis communities within each population subgroup. While Walktrap attempts to find densely connected subgraphs, via random walks, label propagation utilizes a majority voting technique where at each iteration a diagnosis node adopts the label that most of its neighbors currently have.

## Additional Information

**How to cite this article**: Feldman, K. *et al*. Insights into Population Health Management Through Disease Diagnoses Networks. *Sci. Rep.* **6**, 30465; doi: 10.1038/srep30465 (2016).

## Supplementary Material

Supplementary Information

## Figures and Tables

**Table 1:** Ranked list of edges where Low Income (LI) population is over-represented in comparison to High Income (HI) population.

Rank	Edge Pair	Count		Normalized		Fold-change
		HI	LI	HI	LI	
1	295 - Schizophrenic disorders	26,603	110,521	0.00285	0.00894	3.135
	305 - Nondependent abuse of drugs					
2	295 - Schizophrenic disorders	20,198	74,082	0.00216	0.00599	2.768
	250 - Diabetes mellitus					
3	401 - Essential hypertension	31,180	113,647	0.00334	0.00919	2.750
	295 - Schizophrenic disorders					
4	070 - Viral hepatitis	30,753	108,271	0.0033	0.00875	2.657
	305 - Nondependent abuse of drugs					
5	403 - Hypertensive chronic kidney disease	23,494	81,366	0.00252	0.00658	2.613
	305 - Nondependent abuse of drugs					
6	304 - Drug dependence	9,669	33,232	0.00104	0.00269	2.593
	493 - Asthma					
7	345 - Epilepsy and recurrent seizures	11,249	37,667	0.00121	0.00305	2.527
	305 - Nondependent abuse of drugs					
8	585 - Chronic kidney disease (CKD)	22,892	75,822	0.00245	0.00613	2.499
	305 - Nondependent abuse of drugs					
9	278 - Overweight, obesity and other hyperalimentation	9,576	31,602	0.00103	0.00256	2.490
	295 - Schizophrenic disorders					
10	648 - Other current conditions in the mother classifiable elsewhere but complicating pregnancy, childbirth, or the puerperium	17,608	57,798	0.00189	0.00467	2.477
	305 - Nondependent abuse of drugs					

**Table 2:** Ranked list of edges where High Income (HI) population is over-represented in comparison to Low Income (LI) population.

Rank	Edge Pair	Count		Normalized		Fold-change
		HI	LI	HI	LI	
1	244 - Acquired hypothyroidism	36,054	15,020	0.00386	0.00121	3.181
	648 - Other current conditions in the mother classifiable elsewhere but complicating pregnancy, childbirth, or the puerperium					
2	659 - Other indications for care or intervention related to labor and delivery, not elsewhere classified	139,723	63,894	0.01497	0.00517	2.898
	664 - Trauma to perineum and vulva during delivery					
3	654 - Abnormality of organs and soft tissues of pelvis	86,596	47,674	0.00928	0.00385	2.407
	659 - Other indications for care or intervention related to labor and delivery, not elsewhere classified					
4	652 - Malposition and malpresentation of fetus	39,193	21,995	0.0042	0.00178	2.361
	659 - Other indications for care or intervention related to labor and delivery, not elsewhere classified					
5	663 - Umbilical cord complications	113,957	66,694	0.01221	0.00539	2.264
	659 - Other indications for care or intervention related to labor and delivery, not elsewhere classified					
6	663 - Umbilical cord complications	134,474	80,865	0.01441	0.00654	2.204
	664 - Trauma to perineum and vulva during delivery					
7	660 - Obstructed labor	27,094	16,627	0.0029	0.00134	2.160
	659 - Other indications for care or intervention related to labor and delivery, not elsewhere classified					
8	645 - Late pregnancy	57,453	37,220	0.00616	0.00301	2.046
	659 - Other indications for care or intervention related to labor and delivery, not elsewhere classified					
9	656 - Other known or suspected fetal and placental problems affecting management of mother	40,455	26,209	0.00433	0.00212	2.046
	664 - Trauma to perineum and vulva during delivery					
10	661 - Abnormality of forces of labor	27,986	18,203	0.003	0.00147	2.038
	664 - Trauma to perineum and vulva during delivery					

**Table 3:** Betweenness-Centrality Rankings on unmodified High and Low income population networks.

Rank	Low Income Population			High Income Population		
	ICD Code	Betweenness Centrality	Diagnosis Name	ICD Code	Betweenness Centrality	Diagnosis Name
1	250	0.300	Diabetes mellitus	401	0.295	Essential hypertension
2	401	0.210	Essential hypertension	427	0.162	Cardiac dysrhythmias
3	305	0.102	Nondependent abuse of drugs	272	0.128	Disorders of lipoid metabolism
4	276	0.070	Disorders of fluid, electrolyte, and acid-base	285	0.121	Other and unspecified anemias
5	496	0.047	Chronic airway obstruction not elsewhere classified	244	0.094	Acquired hypothyroidism
6	285	0.041	Other and unspecified anemias	648	0.081	Other current conditions in the mother classifiable elsewhere but complicating pregnancy, childbirth, or the puerperium
7	428	0.038	Heart failure	276	0.080	Disorders of fluid electrolyte, and acid-base balance
8	278	0.028	Overweight, obesity and other hyperalimentatio	311	0.057	Depressive disorder not elsewhere classified
9	414	0.026	Other forms of chronic ischemic heart disease	424	0.028	Other diseases of endocardium
10	599	0.020	Other disorders of urethra and urinary tract	733	0.026	Other disorders of bone and cartilage

**Table 4:** Thresholded Betweenness-Centrality Rankings - Low Income Population.

Rank	ICD Code	Betweenness Centrality	Diagnosis Name	ICD Code	Betweenness Centrality	Diagnosis Name
	*Threshold* - *1*.*25*			*Threshold* - *1*.*50*		
1	305	0.218	Nondependent abuse of drugs	305	0.200	Nondependent abuse of drugs
2	250	0.179	Diabetes mellitus	250	0.045	Diabetes mellitus
3	401	0.036	Essential hypertension	491	0.012	Chronic bronchitis
4	496	0.034	Chronic airway obstruction	401	0.012	Essential hypertension
5	571	0.025	Chronic liver disease and cirrhosis	295	0.012	Schizophrenic disorders
	*Threshold* - *1*.*75*			*Threshold* - *2*.*0*		
1	305	0.070	Nondependent abuse of drugs	305	0.020	Nondependent abuse of drugs
2	250	0.012	Diabetes mellitus	295	0.007	Schizophrenic disorders
3	70	0.010	Viral hepatitis	491	0.005	Chronic bronchitis
4	491	0.008	Chronic bronchitis	70	0.004	Viral hepatitis
5	295	0.006	Schizophrenic disorders	250	0.001	Diabetes mellitus

**Table 5:** Thresholded Betweenness-Centrality Rankings - High Income Population.

Rank	ICD Code	Betweenness Centrality	Diagnosis Name	ICD Code	Betweenness Centrality	Diagnosis Name
	*Threshold* - *1*.*25*			*Threshold* - *1*.*50*		
1	427	0.068	Cardiac dysrhythmias	244	0.027	Acquired hypothyroidism
2	244	0.065	Acquired hypothyroidism	648	0.022	Other current conditions in the mother classifiable elsewhere but complicating pregnancy, childbirth, or the puerperium
3	424	0.060	Other diseases of endocardium	424	0.019	Other diseases of endocardium
4	272	0.055	Disorders of lipoid metabolism	427	0.017	Cardiac dysrhythmias
	*Threshold* - *1*.*75*			*Threshold* - *2*.*0*		
5	648	0.047	Other current conditions in the mother classifiable elsewhere but complicating pregnancy, childbirth, or the puerperium	272	0.014	Disorders of lipoid metabolism
1	664	0.001	Trauma to perineum and vulva during delivery	659	0.001	Other indications for care or intervention related to labor and delivery not elsewhere classified
2	659	0.001	Other indications for care or intervention related to labor and delivery not elsewhere classified	664	0.0006	Trauma to perineum and vulva during delivery
3	648	0.0005	Other current conditions in the mother classifiable elsewhere but complicating pregnancy, childbirth, or the puerperium			
4	663	0.0001	Umbilical cord complications			
5	645	0.00001	Late pregnancy			
