# Supplementary material for: Insights into Population Health Management Through Disease Diagnoses Networks
Source: Sci Rep. 2016 Jul 27;6:30465. doi: 10.1038/srep30465 (PMC4962032; doi:10.1038/srep30465)
Supplement: Supplementary Information [file srep30465-s1.pdf]

Insights into Population Health Management Through Disease Diagnoses Networks

Keith Feldman[1,2], Gregor Stiglic[3,4], Dipanwita Dasgupta[1,2], Mark Kricheff[5], Zoran Obradovic[6], and Nitesh V. Chawla[1,2,7,*]

[1]University of Notre Dame, Computer Science and Engineering, Notre Dame, 46556, USA
[2]University of Notre Dame, Interdisciplinary Center for Network Science and Applications (iCeNSA), Notre Dame, 46556, USA
[3]University of Maribor, Health Sciences, Maribor, 2000, Slovenia
[4]University of Maribor, Electrical Engineering and Computer Science, Maribor, 2000, Slovenia
[5]St.Joseph's Regional Medical Center, Mishawaka, 46544, USA
[6]Temple University, Data Analytics and Biomedical Informatics Center, Philadelphia , 19122 , USA
[7]Wroclaw University of Technology, Wrocław ,50-370, Poland
[*]nchawla@nd.edu

Supplementary Information

Data and Methods

In order to demonstrate the generalizability of the network analysis technique detailed in this work we have extended the analysis to investigate over-represented diagnoses between subgroups for a general population.

Data

We will again utilize the full set of 21,662,600 patient hospitalization records drawn from the Nationwide Inpatient Sample (NIS) provided by the Healthcare Cost and Utilization Project (HCUP). As in the main text the data is then partitioned into the two base populations of 9,306,956 high income and 12,355,644 low income records based on the income data provided by NIS. The low income population is determined by those patients in the lowest median income quartile, while the high income population represents those in highest median income quartile.

We then take it a step further breaking each population down into subgroups based on the individual's insurance type. For this example we will focus on the Medicaid and Private insurance plans subgroups, however the analysis could be easily adapted to other subgroups including any of the 6 possible insurance types. After partitioning we are left with 2,554,812 Medicaid, 2,809,511 Private insurance records within the low income population, and 624,003 Medicaid, 4,306,029 Private insurance records within the high income population.

Methods

Utilizing these populations subgroups disease networks were constructed, the process including the edge normalization function can be found in the Methods sections of the main text. Following the same methodology found in the main text for the general populations the ratios between the low and high income insurance plans were computed to identify those diagnoses which occur in greater proportion amongst each of the population subgroups

Results

Using the visualization transformation detailed in the Methods section of the main text the resulting networks for the Medicaid and Private insurance plans can be found in Supplementary Figures 1 and 2 respectively. The visualization threshold used for each network can be at the top of figure.

As with the general population we then identified the top 10 more over-represented edges between each insurance subgroup for both the high and low income populations. These can be found in Supplementary Tables 1, 2, 3 and 4

Discussion

As with the complete high income population group utilized in the main paper, the high income groups for both insurance groups demonstrate over-represented conditions focused primarily on pregnancy and birth related conditions. However, there is an important difference between the diagnoses in Medicaid and Private insurance subgroups. The majority of the conditions identified within the Medicaid subgroup are those which would be diagnosed at the time of birth, such as umbilical cord complications (663) or obstructed labor (660). However the conditions comprising the high income private insurance subgroup are those likely diagnosed during prenatal care visits, such as suspected fetal and placental problems affecting management of mother (656), and hypothyroidism (244). As noted in the main paper it is well established that due to the cost prenatal care visits, as well as limited access to resources lower socioeconomic populations often times fail to receive prenatal care at the appropriate times, if at all.[1] This differentiation further exemplifies the benefit of this network technique to isolate a diagnosis category (pregnancy) to target particular subgroups (insurance type). Continuing with an analysis of the diagnoses comprising the Medicaid and Private subgroups within the low income population group, we present additional potentially interesting deviations. Examining the Private Insurance subgroup we find the majority of conditions relate to chronic diseases such as diabetes, hypertension, heart disease, and bronchitis. Many of which have been tied to individuals with lower socioeconomic status.[2–4]

The low income population within Medicare subgroup however was comprised primarily of the diagnosis Human immunodeficiency (042). This is a motivating distinction. It has been well documented that individuals in lower socioeconomic conditions are at higher risk for HIV.[5] Prior studies have even gone so far as to shown that the correlation may be bi-directional, that HIV has a negative impact on individual's socioeconomic status.[6,7] While both sets of conditions are linked to low socioeconomic status the proportions of HIV-infected adults with Medicaid insurance was just over double that who carried private insurance.[8]

Conclusion

The work presented in this supplementary analysis demonstrates that the populations on which the fold-change metric can be effectively applied are not limited to a single partitioning, such as income. Rather, the population subgroups can be derived from more complex multi-level partitionings. This increased specificity combined with the expansive set of health data collected around the world, can help in yielding insights into the predispositions and current health trends for increasingly targeted populations, particularly for those whose patients may be widely dispersed or few in number. The utilization of these insights is the logical next step for any physician or community health worker attempting to employ this form of informatics for targeted care and resource management, given socio-economic considerations.

References

1. Sword, W. A socio-ecological approach to understanding barriers to prenatal care for women of low income. *Journal of Advanced Nursing* **29**, 1170–1177 (1999).

2. Robbins, J. M., Vaccarino, V., Zhang, H. & Kasl, S. V. Socioeconomic status and diagnosed diabetes incidence. *Diabetes research and clinical practice* **68**, 230–236 (2005).

3. Franks, P., Winters, P. C., Tancredi, D. J. & Fiscella, K. A. Do changes in traditional coronary heart disease risk factors over time explain the association between socio-economic status and coronary heart disease? *BMC cardiovascular disorders* **11**, 28 (2011).

4. Hawkins, N. M., Jhund, P. S., McMurray, J. J. & Capewell, S. Heart failure and socioeconomic status: accumulating evidence of inequality. *European journal of heart failure* **14**, 138–146 (2012).

5. Perry, M. J. Gender, race and economic perspectives on the social epidemiology of hiv infection: implications for prevention. *The Journal of Primary Prevention* **19**, 97–104 (1998).

6. Dray-Spira, R., Lert, F., Marimoutou, C., Bouhnik, A.-D. & Obadia, Y. Socio-economic conditions, health status and employment among persons living with hiv/aids in france in 2001. *AIDS care* **15**, 739–748 (2003).

7. Rabkin, J. G., McElhiney, M., Ferrando, S. J., Van Gorp, W. & Lin, S. H. Predictors of employment of men with hiv/aids: a longitudinal study. *Psychosomatic Medicine* **66**, 72–78 (2004).

8. Yehia, B. R. *et al.* Health insurance coverage for persons in hiv care, 2006–2012. *JAIDS Journal of Acquired Immune Deficiency Syndromes* **67**, 102–106 (2014).

		Count		Normalized		
**Rank**	**Edge Pair**	**Hi**	**LI**	**HI**	**LI**	**Fold-Change**
1	042 - Human immunodeficiency virus [HIV] disease<br>304 - Drug dependence	87	1262	0.00115	0.00356	3.087
2	042 - Human immunodeficiency virus [HIV] disease<br>486 - Pneumonia, organism unspecified	95	1349	0.00126	0.0038	3.022
3	042 - Human immunodeficiency virus [HIV] disease<br>285 - Other and unspecified anemias	147	1902	0.00195	0.00536	2.754
4	042 - Human immunodeficiency virus [HIV] disease<br>112 - Candidiasis	141	1816	0.00187	0.00512	2.741
5	042 - Human immunodeficiency virus [HIV] disease<br>401 - Essential hypertension	91	1059	0.00121	0.00299	2.477
6	042 - Human immunodeficiency virus [HIV] disease<br>305 - Nondependent abuse of drugs	184	2035	0.00244	0.00574	2.354
7	491 - Chronic bronchitis<br>786 - Symptoms involving respiratory<br>system and other chest symptoms	82	897	0.00109	0.00253	2.328
8	042 - Human immunodeficiency virus [HIV] disease<br>276 - Disorders of fluid, electrolyte,<br>and acid-base balance	224	2296	0.00297	0.00647	2.18151
9	626 - Disorders of menstruation and<br>other abnormal bleeding from female genital tract<br>625 - Pain and other symptoms<br>associated with female genital organs	97	975	0.00128	0.00275	2.13928
10	042 - Human immunodeficiency virus [HIV] disease<br>070 - Viral hepatitis	175	1725	0.00232	0.00486	2.0979

Table 1. Medicaid: Ranked list of edges where the low income (LI) population is over-represented in comparison to the high income (HI) population

		Count		*Normalized*		
**Rank**	**Edge Pair**	**HI**	**LI**	**HI**	**LI**	**Fold-Change**
1	343 - Infantile cerebral palsy<br>780 - General symptoms	186	363	0.00246	0.00102	2.408
2	652 - Malposition and malpresentation of fetus<br>664 - Trauma to perineum and vulva during delivery	177	398	0.00234	0.00112	2.090
3	645 - Late pregnancy<br>663 - Umbilical cord complications	768	1798	0.01017	0.00507	2.007
4	665 - Other obstetrical trauma<br>664 - Trauma to perineum and vulva during delivery	305	740	0.00404	0.00209	1.937
5	507 - Pneumonitis due to solids and liquids<br>276 - Disorders of fluid, electrolyte, and acid-base balance	383	930	0.00507	0.00262	1.935
6	645 - Late pregnancy<br>660 - Obstructed labor	227	552	0.00301	0.00156	1.932
7	665 - Other obstetrical trauma<br>659 - Other indications for care or intervention<br>related to labor and delivery, not elsewhere classified	236	589	0.00313	0.00166	1.883
8	659 - Other indications for care or intervention<br>related to labor and delivery, not elsewhere classified<br>664 - Trauma to perineum and vulva during delivery	1296	3249	0.01717	0.00916	1.874
9	507 - Pneumonitis due to solids and liquids<br>250 - Diabetes mellitus	161	406	0.00213	0.00114	1.863
10	294 - Persistent mental disorders<br>due to conditions classified elsewhere<br>331 - Other cerebral degenerations	184	465	0.00244	0.00131	1.859

Table 2. Medicaid: Ranked list of edges where the high income (HI) population is over-represented in comparison to the low income (LI) population

		Count		*Normalized*		
**Rank**	**Edge Pair**	**HI**	**LI**	**HI**	**LI**	**Fold-Change**
1	402 - Hypertensive heart disease<br>428 - Heart failure	707	1094	0.00112	0.00278	2.477
2	402 - Hypertensive heart disease<br>250 - Diabetes mellitus	862	1267	0.00137	0.00322	2.352
3	491 - Chronic bronchitis<br>250 - Diabetes mellitus	1234	1711	0.00196	0.00435	2.219
4	070 - Viral hepatitis<br>305 - Nondependent abuse of drugs	991	1344	0.00157	0.00342	2.171
5	716 - Other and unspecified arthropathies<br>250 - Diabetes mellitus	698	941	0.00111	0.00239	2.158
6	428 - Heart failure<br>715 - Osteoarthrosis and allied disorders	768	1006	0.00122	0.00256	2.096
7	491 - Chronic bronchitis<br>401 - Essential hypertension	2593	3378	0.00412	0.00859	2.085
8	491 - Chronic bronchitis<br>428 - Heart failure	1388	1789	0.00221	0.00455	2.063
9	428 - Heart failure<br>305 - Nondependent abuse of drugs	1957	2501	0.00311	0.00636	2.045
10	496 - Chronic airway obstruction, not elsewhere classified<br>715 - Osteoarthrosis and allied disorders	775	989	0.00123	0.00251	2.042

Table 3. Private Insurance: Ranked list of edges where the low income (LI) population is over-represented in comparison to the high income (HI) population

		Count		*Normalized*		
**Rank**	**Edge Pair**	**HI**	**LI**	**HI**	**LI**	**Fold-Change**
1	244 - Acquired hypothyroidism<br>648 - Other current conditions in the mother classifiable elsewhere, but complicating pregnancy, childbirth, or the puerperium	3818	765	0.00607	0.00195	3.118
2	664 - Trauma to perineum and vulva during delivery<br>659 - Other indications for care or intervention related to labor and delivery, not elsewhere classified	15102	3175	0.02399	0.00807	2.972
3	424 - Other diseases of endocardium<br>648 - Other current conditions in the mother classifiable elsewhere, but complicating pregnancy, childbirth, or the puerperium	2127	466	0.00338	0.00118	2.852
4	652 - Malposition and malpresentation of fetus<br>659 - Other indications for care or intervention related to labor and delivery, not elsewhere classified	4411	1039	0.00701	0.00264	2.653
5	663 - Umbilical cord complications<br>659 - Other indications for care or intervention related to labor and delivery, not elsewhere classified	13209	3238	0.02099	0.00823	2.549
6	654 - Abnormality of organs and soft tissues of pelvis<br>659 - Other indications for care or intervention related to labor and delivery, not elsewhere classified	8579	2155	0.01363	0.00548	2.487
7	664 - Trauma to perineum and vulva during delivery<br>656 - Other known or suspected fetal and placental problems affecting management of mother	5157	1296	0.00819	0.0033	2.486
8	645 - Late pregnancy<br>659 - Other indications for care or intervention related to labor and delivery, not elsewhere classified	5761	1450	0.00915	0.00369	2.482
9	652 - Malposition and malpresentation of fetus<br>663 - Umbilical cord complications	2535	643	0.00403	0.00163	2.463
10	663 - Umbilical cord complications<br>664 - Trauma to perineum and vulva during delivery	15796	4066	0.0251	0.01034	2.427

Table 4. Private Insurance: Ranked list of edges where the high income (HI) population is over-represented in comparison to the low income (LI) population

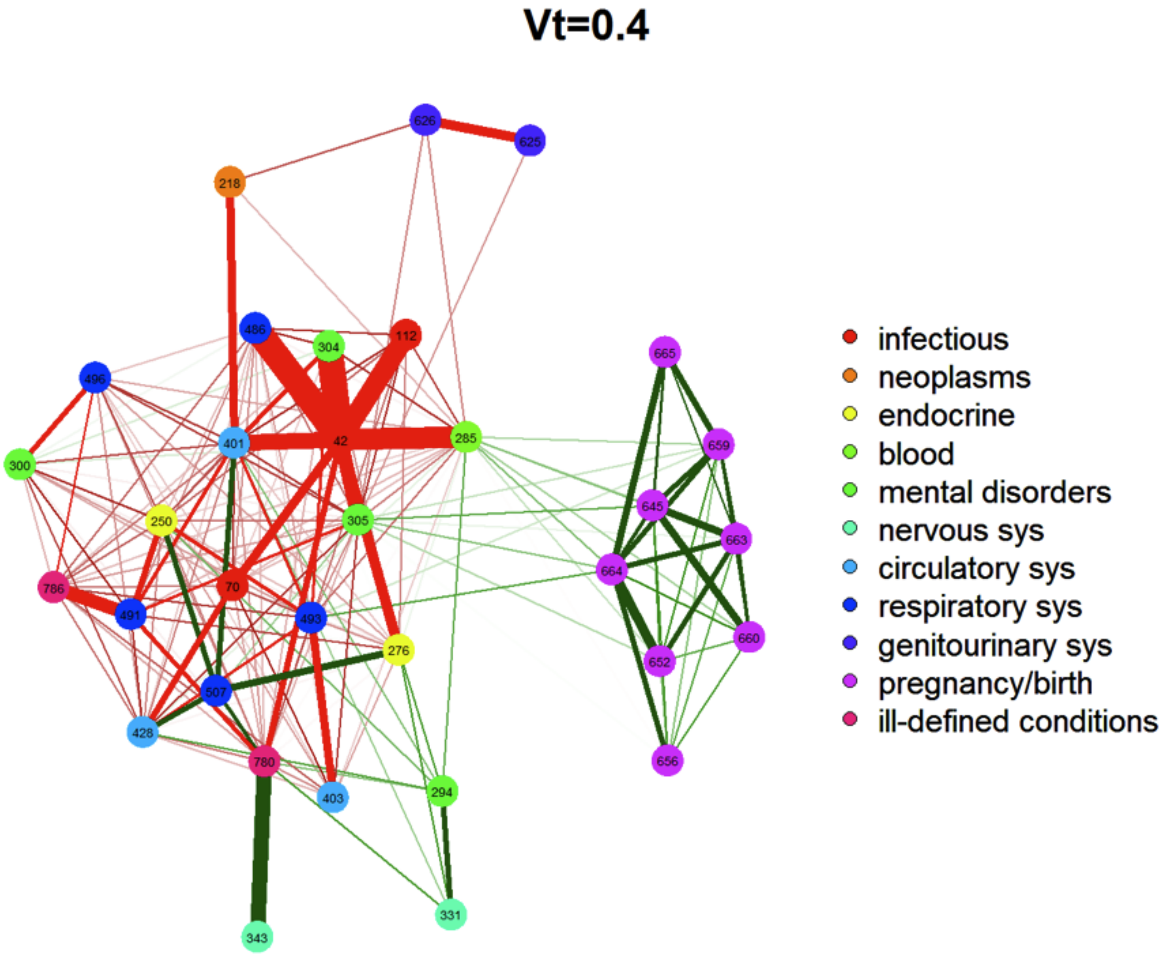

Figure 1. Network representation of over-represented Medicaid diagnoses compared to Private Insurance

Cluster Number	ICD Code	Diagnosis Name
0	512	Pneumothorax and air leak
0	515	Postinflammatory pulmonary fibrosis
0	518	Other diseases of lung
0	8	Intestinal infections due to other organisms
0	530	Diseases of esophagus
0	531	Gastric ulcer
0	532	Duodenal ulcer
0	535	Gastritis and duodenitis
0	537	Other disorders of stomach and duodenum
0	38	Septicemia
0	552	Other hernia of abdominal cavity
0	41	Bacterial infection in conditions classified elsewhere and of unspecified site
0	555	Regional enteritis
0	557	Vascular insufficiency of intestine
0	558	Other and unspecified noninfectious gastroenteritis and colitis

0	560	Intestinal obstruction without mention of hernia
0	562	Diverticula of intestine
0	564	Functional digestive disorders
0	53	Herpes zoster
0	567	Peritonitis and retroperitoneal infections
0	568	Other disorders of peritoneum
0	569	Other disorders of intestine
0	570	Acute and subacute necrosis of liver
0	571	Chronic liver disease and cirrhosis
0	574	Cholelithiasis
0	576	Other disorders of biliary tract
0	577	Diseases of pancreas
0	578	Gastrointestinal hemorrhage
0	583	Nephritis and nephropathy
0	584	Acute kidney failure
0	585	Chronic kidney disease CKD)
0	590	Infections of kidney
0	591	Hydronephrosis
0	592	Calculus of kidney and ureter
0	593	Other disorders of kidney and ureter
0	596	Other disorders of bladder
0	599	Other disorders of urethra and urinary tract
0	600	Hyperplasia of prostate
0	357	Inflammatory and toxic neuropathy
0	924	Contusion of lower limb and of other and unspecified sites
0	784	Symptoms involving head and neck
0	618	Genital prolapse
0	786	Symptoms involving respiratory system and other chest symptoms
0	112	Candidiasis
0	625	Pain and other symptoms associated with female genital organs
0	275	Disorders of mineral metabolism
0	276	Disorders of fluid
0	278	Overweight
0	710	Diffuse diseases of connective tissue
0	682	Other cellulitis and abscess
0	174	Malignant neoplasm of female breast
0	696	Psoriasis and similar disorders
0	185	Malignant neoplasm of prostate
0	799	Other ill-defined and unknown causes of morbidity and mortality
0	714	Rheumatoid arthritis and other inflammatory polyarthropathies
0	707	Chronic ulcer of skin
0	196	Secondary and unspecified malignant neoplasm of lymph nodes
0	197	Secondary malignant neoplasm of respiratory and digestive systems
0	198	Secondary malignant neoplasm of other specified sites
0	199	Malignant neoplasm without specification of site
0	202	Other malignant neoplasms of lymphoid and histiocytic tissue
0	715	Osteoarthrosis and allied disorders
0	719	Other and unspecified disorders of joint
0	721	Spondylosis and allied disorders
0	722	Intervertebral disc disorders
0	211	Benign neoplasm of other parts of digestive system
0	724	Other and unspecified disorders of back

0	726	Peripheral enthesopathies and allied syndromes
0	727	Other disorders of synovium
0	728	Disorders of muscle
0	729	Other disorders of soft tissues
0	733	Other disorders of bone and cartilage
0	805	Fracture of vertebral column without mention of spinal cord injury
0	737	Curvature of spine
0	860	Traumatic pneumothorax and hemothorax
0	238	Neoplasm of uncertain behavior of other and unspecified sites and tissues
0	242	Thyrotoxicosis with or without goiter
0	244	Acquired hypothyroidism
0	553	Other hernia of abdominal cavity without mention of obstruction or gangrene
0	250	Diabetes mellitus
0	253	Disorders of the pituitary gland and its hypothalamic control
0	255	Disorders of adrenal glands
0	263	Other and unspecified protein-calorie malnutrition
0	812	Fracture of humerus
0	780	General symptoms
0	781	Symptoms involving nervous and musculoskeletal systems
0	782	Symptoms involving skin and other integumentary tissue
0	783	Symptoms concerning nutrition
0	272	Disorders of lipoid metabolism
0	785	Symptoms involving cardiovascular system
0	274	Gout
0	787	Symptoms involving digestive system
0	788	Symptoms involving urinary system
0	789	Other symptoms involving abdomen and pelvis
0	790	Nonspecific findings on examination of blood
0	280	Iron deficiency anemias
0	794	Nonspecific abnormal results of function studies
0	284	Aplastic anemia and other bone marrow failure syndromes
0	285	Other and unspecified anemias
0	286	Coagulation defects
0	287	Purpura and other hemorrhagic conditions
0	288	Diseases of white blood cells
0	289	Other diseases of blood and blood-forming organs
0	290	Dementias
0	291	Alcohol-induced mental disorders
0	292	Drug-induced mental disorders
0	293	Transient mental disorders due to conditions classified elsewhere
0	294	Persistent mental disorders due to conditions classified elsewhere
0	807	Fracture of ribs)
0	296	Episodic mood disorders
0	300	Anxiety
0	303	Alcohol dependence syndrome
0	820	Fracture of neck of femur
0	311	Depressive disorder
0	824	Fracture of ankle
0	327	Organic sleep disorders
0	331	Other cerebral degenerations
0	332	”Parkinsons disease
0	333	Other extrapyramidal disease and abnormal movement disorders

0	338	Pain
0	340	Multiple sclerosis
0	342	Hemiplegia and hemiparesis
0	346	Migraine
0	348	Other conditions of brain
0	355	Mononeuritis of lower limb
0	356	Hereditary and idiopathic peripheral neuropathy
0	486	Pneumonia
0	873	Other open wound of head
0	362	Other retinal disorders
0	365	Glaucoma
0	368	Visual disturbances
0	386	Vertiginous syndromes and other disorders of vestibular system
0	389	Hearing loss
0	396	Diseases of mitral and aortic valves
0	397	Diseases of other endocardial structures
0	398	Other rheumatic heart disease
0	401	Essential hypertension
0	403	Hypertensive chronic kidney disease
0	153	Malignant neoplasm of colon
0	920	Contusion of face
0	410	Acute myocardial infarction
0	411	Other acute and subacute forms of ischemic heart disease
0	412	Old myocardial infarction
0	413	Angina pectoris
0	414	Other forms of chronic ischemic heart disease
0	415	Acute pulmonary heart disease
0	416	Chronic pulmonary heart disease
0	423	Other diseases of pericardium
0	424	Other diseases of endocardium
0	425	Cardiomyopathy
0	426	Conduction disorders
0	427	Cardiac dysrhythmias
0	428	Heart failure
0	429	Ill-defined descriptions and complications of heart disease
0	431	Intracerebral hemorrhage
0	433	Occlusion and stenosis of precerebral arteries
0	434	Occlusion of cerebral arteries
0	435	Transient cerebral ischemia
0	437	Other and ill-defined cerebrovascular disease
0	438	Late effects of cerebrovascular disease
0	440	Atherosclerosis
0	441	Aortic aneurysm and dissection
0	443	Other peripheral vascular disease
0	453	Other venous embolism and thrombosis
0	455	Hemorrhoids
0	969	Poisoning by psychotropic agents
0	458	Hypotension
0	459	Other disorders of circulatory system
0	162	Malignant neoplasm of trachea
0	473	Chronic sinusitis
0	477	Allergic rhinitis

0	478	Other diseases of upper respiratory tract
0	482	Other bacterial pneumonia
0	995	Certain adverse effects not elsewhere classified
0	996	Complications peculiar to certain specified procedures
0	997	Complications affecting specified body systems
0	998	Other complications of procedures
0	492	Emphysema
0	493	Asthma
0	496	Chronic airway obstruction
0	507	Pneumonitis due to solids and liquids
0	511	Pleurisy
1	54	Herpes simplex
1	647	Infectious and parasitic conditions in the mother classifiable elsewhere
2	614	Inflammatory disease of ovary
2	616	Inflammatory disease of cervix
2	617	Endometriosis
2	620	Noninflammatory disorders of ovary
2	626	Disorders of menstruation and other abnormal bleeding from female genital tract
2	218	Uterine leiomyoma
3	641	Antepartum hemorrhage
3	642	Hypertension complicating pregnancy
3	644	Early or threatened labor
3	645	Late pregnancy
3	646	Other complications of pregnancy
3	648	Other current conditions in the mother classifiable elsewhere
3	652	Malposition and malpresentation of fetus
3	654	Abnormality of organs and soft tissues of pelvis
3	656	Other known or suspected fetal and placental problems affecting management of mother
3	658	Other problems associated with amniotic cavity and membranes
3	659	Other indications for care or intervention related to labor and delivery
3	660	Obstructed labor
3	661	Abnormality of forces of labor
3	663	Umbilical cord complications
3	664	Trauma to perineum and vulva during delivery
3	665	Other obstetrical trauma
3	666	Postpartum hemorrhage
3	669	Other complications of labor and delivery

Table 5. High Income Community Detection - Label Propagation

Cluster Number	ICD Code	Diagnosis Name
0	515	Postinflammatory pulmonary fibrosis
0	518	Other diseases of lung
0	519	Other diseases of respiratory system
0	8	Intestinal infections due to other organisms
0	530	Diseases of esophagus
0	531	Gastric ulcer
0	532	Duodenal ulcer
0	533	Peptic ulcer
0	535	Gastritis and duodenitis
0	536	Disorders of function of stomach

0	38	Septicemia
0	41	Bacterial infection in conditions classified elsewhere and of unspecified site
0	558	Other and unspecified noninfectious gastroenteritis and colitis
0	560	Intestinal obstruction without mention of hernia
0	562	Diverticula of intestine
0	564	Functional digestive disorders
0	569	Other disorders of intestine
0	571	Chronic liver disease and cirrhosis
0	572	Liver abscess and sequelae of chronic liver disease
0	573	Other disorders of liver
0	574	Cholelithiasis
0	575	Other disorders of gallbladder
0	577	Diseases of pancreas
0	578	Gastrointestinal hemorrhage
0	70	Viral hepatitis
0	583	Nephritis and nephropathy
0	584	Acute kidney failure
0	585	Chronic kidney disease CKD)
0	588	Disorders resulting from impaired renal function
0	590	Infections of kidney
0	592	Calculus of kidney and ureter
0	593	Other disorders of kidney and ureter
0	596	Other disorders of bladder
0	599	Other disorders of urethra and urinary tract
0	600	Hyperplasia of prostate
0	784	Symptoms involving head and neck
0	614	Inflammatory disease of ovary
0	273	Disorders of plasma protein metabolism
0	620	Noninflammatory disorders of ovary
0	274	Gout
0	110	Dermatophytosis
0	112	Candidiasis
0	625	Pain and other symptoms associated with female genital organs
0	626	Disorders of menstruation and other abnormal bleeding from female genital tract
0	787	Symptoms involving digestive system
0	788	Symptoms involving urinary system
0	642	Hypertension complicating pregnancy
0	790	Nonspecific findings on examination of blood
0	646	Other complications of pregnancy
0	647	Infectious and parasitic conditions in the mother classifiable elsewhere
0	648	Other current conditions in the mother classifiable elsewhere
0	649	Other conditions or status of the mother complicating pregnancy
0	654	Abnormality of organs and soft tissues of pelvis
0	659	Other indications for care or intervention related to labor and delivery
0	281	Other deficiency anemias
0	153	Malignant neoplasm of colon
0	162	Malignant neoplasm of trachea
0	710	Diffuse diseases of connective tissue
0	681	Cellulitis and abscess of finger and toe
0	682	Other cellulitis and abscess
0	287	Purpura and other hemorrhagic conditions
0	707	Chronic ulcer of skin

0	197	Secondary malignant neoplasm of respiratory and digestive systems
0	198	Secondary malignant neoplasm of other specified sites
0	199	Malignant neoplasm without specification of site
0	714	Rheumatoid arthritis and other inflammatory polyarthropathies
0	715	Osteoarthrosis and allied disorders
0	716	Other and unspecified arthropathies
0	719	Other and unspecified disorders of joint
0	721	Spondylosis and allied disorders
0	722	Intervertebral disc disorders
0	211	Benign neoplasm of other parts of digestive system
0	724	Other and unspecified disorders of back
0	728	Disorders of muscle
0	729	Other disorders of soft tissues
0	730	Osteomyelitis
0	731	Osteitis deformans and osteopathies associated with other disorders classified elsewhere
0	733	Other disorders of bone and cartilage
0	242	Thyrotoxicosis with or without goiter
0	723	Other disorders of cervical region
0	244	Acquired hypothyroidism
0	553	Other hernia of abdominal cavity without mention of obstruction or gangrene
0	250	Diabetes mellitus
0	251	Other disorders of pancreatic internal secretion
0	263	Other and unspecified protein-calorie malnutrition
0	266	Deficiency of B-complex components
0	780	General symptoms
0	781	Symptoms involving nervous and musculoskeletal systems
0	782	Symptoms involving skin and other integumentary tissue
0	783	Symptoms concerning nutrition
0	272	Disorders of lipoid metabolism
0	785	Symptoms involving cardiovascular system
0	786	Symptoms involving respiratory system and other chest symptoms
0	275	Disorders of mineral metabolism
0	276	Disorders of fluid
0	789	Other symptoms involving abdomen and pelvis
0	278	Overweight
0	280	Iron deficiency anemias
0	998	Other complications of procedures
0	794	Nonspecific abnormal results of function studies
0	284	Aplastic anemia and other bone marrow failure syndromes
0	285	Other and unspecified anemias
0	286	Coagulation defects
0	799	Other ill-defined and unknown causes of morbidity and mortality
0	288	Diseases of white blood cells
0	290	Dementias
0	291	Alcohol-induced mental disorders
0	292	Drug-induced mental disorders
0	293	Transient mental disorders due to conditions classified elsewhere
0	294	Persistent mental disorders due to conditions classified elsewhere
0	295	Schizophrenic disorders
0	296	Episodic mood disorders
0	298	Other nonorganic psychoses
0	300	Anxiety

0	301	Personality disorders
0	303	Alcohol dependence syndrome
0	304	Drug dependence
0	305	Nondependent abuse of drugs
0	820	Fracture of neck of femur
0	309	Adjustment reaction
0	311	Depressive disorder
0	327	Organic sleep disorders
0	331	Other cerebral degenerations
0	332	”Parkinsons disease
0	333	Other extrapyramidal disease and abnormal movement disorders
0	337	Disorders of the autonomic nervous system
0	338	Pain
0	342	Hemiplegia and hemiparesis
0	344	Other paralytic syndromes
0	345	Epilepsy and recurrent seizures
0	346	Migraine
0	348	Other conditions of brain
0	355	Mononeuritis of lower limb
0	356	Hereditary and idiopathic peripheral neuropathy
0	357	Inflammatory and toxic neuropathy
0	362	Other retinal disorders
0	365	Glaucoma
0	369	Blindness and low vision
0	389	Hearing loss
0	397	Diseases of other endocardial structures
0	401	Essential hypertension
0	402	Hypertensive heart disease
0	403	Hypertensive chronic kidney disease
0	404	Hypertensive heart and chronic kidney disease
0	218	Uterine leiomyoma
0	410	Acute myocardial infarction
0	411	Other acute and subacute forms of ischemic heart disease
0	412	Old myocardial infarction
0	413	Angina pectoris
0	414	Other forms of chronic ischemic heart disease
0	415	Acute pulmonary heart disease
0	416	Chronic pulmonary heart disease
0	424	Other diseases of endocardium
0	425	Cardiomyopathy
0	426	Conduction disorders
0	427	Cardiac dysrhythmias
0	428	Heart failure
0	429	Ill-defined descriptions and complications of heart disease
0	433	Occlusion and stenosis of precerebral arteries
0	434	Occlusion of cerebral arteries
0	435	Transient cerebral ischemia
0	437	Other and ill-defined cerebrovascular disease
0	438	Late effects of cerebrovascular disease
0	440	Atherosclerosis
0	441	Aortic aneurysm and dissection
0	443	Other peripheral vascular disease

0	444	Arterial embolism and thrombosis
0	447	Other disorders of arteries and arterioles
0	453	Other venous embolism and thrombosis
0	455	Hemorrhoids
0	456	Varicose veins of other sites
0	969	Poisoning by psychotropic agents
0	458	Hypotension
0	459	Other disorders of circulatory system
0	465	Acute upper respiratory infections of multiple or unspecified sites
0	466	Acute bronchitis and bronchiolitis
0	473	Chronic sinusitis
0	477	Allergic rhinitis
0	482	Other bacterial pneumonia
0	995	Certain adverse effects not elsewhere classified
0	996	Complications peculiar to certain specified procedures
0	997	Complications affecting specified body systems
0	486	Pneumonia
0	490	Bronchitis
0	491	Chronic bronchitis
0	492	Emphysema
0	493	Asthma
0	496	Chronic airway obstruction
0	507	Pneumonitis due to solids and liquids
0	511	Pleurisy

Table 6. Low Income Community Detection - Label Propagation

Cluster Number	ICD Code	Diagnosis Name
0	512	Pneumothorax and air leak
0	532	Duodenal ulcer
0	537	Other disorders of stomach and duodenum
0	557	Vascular insufficiency of intestine
0	568	Other disorders of peritoneum
0	576	Other disorders of biliary tract
0	592	Calculus of kidney and ureter
0	174	Malignant neoplasm of female breast
0	202	Other malignant neoplasms of lymphoid and histiocytic tissue
0	253	Disorders of the pituitary gland and its hypothalamic control
0	255	Disorders of adrenal glands
0	289	Other diseases of blood and blood-forming organs
0	293	Transient mental disorders due to conditions classified elsewhere
0	873	Other open wound of head
0	401	Essential hypertension
1	515	Postinflammatory pulmonary fibrosis
1	518	Other diseases of lung
1	8	Intestinal infections due to other organisms
1	530	Diseases of esophagus
1	38	Septicemia
1	41	Bacterial infection in conditions classified elsewhere and of unspecified site
1	560	Intestinal obstruction without mention of hernia
1	562	Diverticula of intestine

1	564	Functional digestive disorders
1	569	Other disorders of intestine
1	571	Chronic liver disease and cirrhosis
1	574	Cholelithiasis
1	577	Diseases of pancreas
1	578	Gastrointestinal hemorrhage
1	584	Acute kidney failure
1	585	Chronic kidney disease CKD
1	591	Hydronephrosis
1	593	Other disorders of kidney and ureter
1	596	Other disorders of bladder
1	599	Other disorders of urethra and urinary tract
1	600	Hyperplasia of prostate
1	357	Inflammatory and toxic neuropathy
1	784	Symptoms involving head and neck
1	786	Symptoms involving respiratory system and other chest symptoms
1	112	Candidiasis
1	275	Disorders of mineral metabolism
1	276	Disorders of fluid
1	278	Overweight
1	682	Other cellulitis and abscess
1	185	Malignant neoplasm of prostate
1	799	Other ill-defined and unknown causes of morbidity and mortality
1	714	Rheumatoid arthritis and other inflammatory polyarthropathies
1	707	Chronic ulcer of skin
1	196	Secondary and unspecified malignant neoplasm of lymph nodes
1	197	Secondary malignant neoplasm of respiratory and digestive systems
1	198	Secondary malignant neoplasm of other specified sites
1	715	Osteoarthrosis and allied disorders
1	719	Other and unspecified disorders of joint
1	721	Spondylosis and allied disorders
1	722	Intervertebral disc disorders
1	211	Benign neoplasm of other parts of digestive system
1	724	Other and unspecified disorders of back
1	728	Disorders of muscle
1	729	Other disorders of soft tissues
1	733	Other disorders of bone and cartilage
1	238	Neoplasm of uncertain behavior of other and unspecified sites and tissues
1	244	Acquired hypothyroidism
1	553	Other hernia of abdominal cavity without mention of obstruction or gangrene
1	250	Diabetes mellitus
1	263	Other and unspecified protein-calorie malnutrition
1	780	General symptoms
1	781	Symptoms involving nervous and musculoskeletal systems
1	782	Symptoms involving skin and other integumentary tissue
1	783	Symptoms concerning nutrition
1	272	Disorders of lipoid metabolism
1	785	Symptoms involving cardiovascular system
1	274	Gout
1	787	Symptoms involving digestive system
1	788	Symptoms involving urinary system
1	789	Other symptoms involving abdomen and pelvis

1	790	Nonspecific findings on examination of blood
1	280	Iron deficiency anemias
1	794	Nonspecific abnormal results of function studies
1	284	Aplastic anemia and other bone marrow failure syndromes
1	285	Other and unspecified anemias
1	286	Coagulation defects
1	287	Purpura and other hemorrhagic conditions
1	288	Diseases of white blood cells
1	290	Dementias
1	292	Drug-induced mental disorders
1	294	Persistent mental disorders due to conditions classified elsewhere
1	300	Anxiety
1	820	Fracture of neck of femur
1	311	Depressive disorder
1	327	Organic sleep disorders
1	331	Other cerebral degenerations
1	332	”Parkinsons disease
1	333	Other extrapyramidal disease and abnormal movement disorders
1	338	Pain
1	346	Migraine
1	348	Other conditions of brain
1	356	Hereditary and idiopathic peripheral neuropathy
1	486	Pneumonia
1	362	Other retinal disorders
1	365	Glaucoma
1	389	Hearing loss
1	396	Diseases of mitral and aortic valves
1	397	Diseases of other endocardial structures
1	398	Other rheumatic heart disease
1	403	Hypertensive chronic kidney disease
1	153	Malignant neoplasm of colon
1	410	Acute myocardial infarction
1	411	Other acute and subacute forms of ischemic heart disease
1	412	Old myocardial infarction
1	413	Angina pectoris
1	414	Other forms of chronic ischemic heart disease
1	415	Acute pulmonary heart disease
1	416	Chronic pulmonary heart disease
1	424	Other diseases of endocardium
1	425	Cardiomyopathy
1	426	Conduction disorders
1	427	Cardiac dysrhythmias
1	428	Heart failure
1	433	Occlusion and stenosis of precerebral arteries
1	434	Occlusion of cerebral arteries
1	435	Transient cerebral ischemia
1	437	Other and ill-defined cerebrovascular disease
1	438	Late effects of cerebrovascular disease
1	440	Atherosclerosis
1	441	Aortic aneurysm and dissection
1	443	Other peripheral vascular disease
1	453	Other venous embolism and thrombosis

1	455	Hemorrhoids
1	458	Hypotension
1	459	Other disorders of circulatory system
1	162	Malignant neoplasm of trachea
1	995	Certain adverse effects not elsewhere classified
1	996	Complications peculiar to certain specified procedures
1	997	Complications affecting specified body systems
1	998	Other complications of procedures
1	492	Emphysema
1	493	Asthma
1	496	Chronic airway obstruction
1	507	Pneumonitis due to solids and liquids
1	511	Pleurisy
2	531	Gastric ulcer
3	535	Gastritis and duodenitis
4	552	Other hernia of abdominal cavity
5	555	Regional enteritis
6	558	Other and unspecified noninfectious gastroenteritis and colitis
7	53	Herpes zoster
8	54	Herpes simplex
8	647	Infectious and parasitic conditions in the mother classifiable elsewhere
9	567	Peritonitis and retroperitoneal infections
10	570	Acute and subacute necrosis of liver
11	583	Nephritis and nephropathy
12	590	Infections of kidney
13	924	Contusion of lower limb and of other and unspecified sites
14	614	Inflammatory disease of ovary
14	617	Endometriosis
14	620	Noninflammatory disorders of ovary
14	626	Disorders of menstruation and other abnormal bleeding from female genital tract
14	218	Uterine leiomyoma
15	616	Inflammatory disease of cervix
16	618	Genital prolapse
16	625	Pain and other symptoms associated with female genital organs
17	641	Antepartum hemorrhage
18	642	Hypertension complicating pregnancy
18	644	Early or threatened labor
18	645	Late pregnancy
18	648	Other current conditions in the mother classifiable elsewhere
18	652	Malposition and malpresentation of fetus
18	654	Abnormality of organs and soft tissues of pelvis
18	656	Other known or suspected fetal and placental problems affecting management of mother
18	658	Other problems associated with amniotic cavity and membranes
18	659	Other indications for care or intervention related to labor and delivery
18	660	Obstructed labor
18	661	Abnormality of forces of labor
18	663	Umbilical cord complications
18	664	Trauma to perineum and vulva during delivery
18	669	Other complications of labor and delivery
19	646	Other complications of pregnancy
20	665	Other obstetrical trauma
21	666	Postpartum hemorrhage

22	710	Diffuse diseases of connective tissue
23	696	Psoriasis and similar disorders
24	199	Malignant neoplasm without specification of site
25	726	Peripheral enthesopathies and allied syndromes
26	727	Other disorders of synovium
27	805	Fracture of vertebral column without mention of spinal cord injury
28	737	Curvature of spine
29	860	Traumatic pneumothorax and hemothorax
30	242	Thyrotoxicosis with or without goiter
31	812	Fracture of humerus
32	291	Alcohol-induced mental disorders
32	296	Episodic mood disorders
32	303	Alcohol dependence syndrome
33	807	Fracture of ribs
34	824	Fracture of ankle
35	340	Multiple sclerosis
36	342	Hemiplegia and hemiparesis
37	355	Mononeuritis of lower limb
38	368	Visual disturbances
39	386	Vertiginous syndromes and other disorders of vestibular system
40	920	Contusion of face
41	423	Other diseases of pericardium
42	429	Ill-defined descriptions and complications of heart disease
43	431	Intracerebral hemorrhage
44	969	Poisoning by psychotropic agents
45	473	Chronic sinusitis
46	477	Allergic rhinitis
47	478	Other diseases of upper respiratory tract
48	482	Other bacterial pneumonia

Table 7. High Income Community Detection - Walktrap

**Cluster Number**	**ICD Code**	**Diagnosis Name**
0	515	Postinflammatory pulmonary fibrosis
0	600	Hyperplasia of prostate
0	788	Symptoms involving urinary system
0	198	Secondary malignant neoplasm of other specified sites
0	714	Rheumatoid arthritis and other inflammatory polyarthropathies
0	721	Spondylosis and allied disorders
0	722	Intervertebral disc disorders
0	733	Other disorders of bone and cartilage
0	250	Diabetes mellitus
0	781	Symptoms involving nervous and musculoskeletal systems
0	998	Other complications of procedures
0	294	Persistent mental disorders due to conditions classified elsewhere
0	305	Nondependent abuse of drugs
0	415	Acute pulmonary heart disease
0	426	Conduction disorders
0	433	Occlusion and stenosis of precerebral arteries
0	997	Complications affecting specified body systems
1	518	Other diseases of lung

1	530	Diseases of esophagus
1	531	Gastric ulcer
1	536	Disorders of function of stomach
1	38	Septicemia
1	41	Bacterial infection in conditions classified elsewhere and of unspecified site
1	560	Intestinal obstruction without mention of hernia
1	562	Diverticula of intestine
1	564	Functional digestive disorders
1	578	Gastrointestinal hemorrhage
1	583	Nephritis and nephropathy
1	584	Acute kidney failure
1	585	Chronic kidney disease CKD
1	588	Disorders resulting from impaired renal function
1	590	Infections of kidney
1	593	Other disorders of kidney and ureter
1	599	Other disorders of urethra and urinary tract
1	784	Symptoms involving head and neck
1	274	Gout
1	112	Candidiasis
1	787	Symptoms involving digestive system
1	790	Nonspecific findings on examination of blood
1	162	Malignant neoplasm of trachea
1	682	Other cellulitis and abscess
1	707	Chronic ulcer of skin
1	715	Osteoarthrosis and allied disorders
1	716	Other and unspecified arthropathies
1	728	Disorders of muscle
1	553	Other hernia of abdominal cavity without mention of obstruction or gangrene
1	263	Other and unspecified protein-calorie malnutrition
1	780	General symptoms
1	782	Symptoms involving skin and other integumentary tissue
1	783	Symptoms concerning nutrition
1	272	Disorders of lipoid metabolism
1	785	Symptoms involving cardiovascular system
1	786	Symptoms involving respiratory system and other chest symptoms
1	275	Disorders of mineral metabolism
1	276	Disorders of fluid
1	789	Other symptoms involving abdomen and pelvis
1	278	Overweight
1	280	Iron deficiency anemias
1	794	Nonspecific abnormal results of function studies
1	285	Other and unspecified anemias
1	799	Other ill-defined and unknown causes of morbidity and mortality
1	288	Diseases of white blood cells
1	290	Dementias
1	300	Anxiety
1	311	Depressive disorder
1	327	Organic sleep disorders
1	331	Other cerebral degenerations
1	342	Hemiplegia and hemiparesis
1	345	Epilepsy and recurrent seizures
1	348	Other conditions of brain

1	357	Inflammatory and toxic neuropathy
1	362	Other retinal disorders
1	403	Hypertensive chronic kidney disease
1	404	Hypertensive heart and chronic kidney disease
1	410	Acute myocardial infarction
1	411	Other acute and subacute forms of ischemic heart disease
1	412	Old myocardial infarction
1	414	Other forms of chronic ischemic heart disease
1	416	Chronic pulmonary heart disease
1	425	Cardiomyopathy
1	428	Heart failure
1	434	Occlusion of cerebral arteries
1	437	Other and ill-defined cerebrovascular disease
1	438	Late effects of cerebrovascular disease
1	440	Atherosclerosis
1	443	Other peripheral vascular disease
1	453	Other venous embolism and thrombosis
1	458	Hypotension
1	459	Other disorders of circulatory system
1	466	Acute bronchitis and bronchiolitis
1	482	Other bacterial pneumonia
1	995	Certain adverse effects not elsewhere classified
1	996	Complications peculiar to certain specified procedures
1	486	Pneumonia
1	491	Chronic bronchitis
1	492	Emphysema
1	493	Asthma
1	496	Chronic airway obstruction
1	511	Pleurisy
2	519	Other diseases of respiratory system
3	8	Intestinal infections due to other organisms
4	532	Duodenal ulcer
5	533	Peptic ulcer
5	273	Disorders of plasma protein metabolism
5	281	Other deficiency anemias
5	710	Diffuse diseases of connective tissue
5	291	Alcohol-induced mental disorders
5	344	Other paralytic syndromes
5	369	Blindness and low vision
5	401	Essential hypertension
5	435	Transient cerebral ischemia
5	473	Chronic sinusitis
6	535	Gastritis and duodenitis
6	455	Hemorrhoids
7	558	Other and unspecified noninfectious gastroenteritis and colitis
7	719	Other and unspecified disorders of joint
7	724	Other and unspecified disorders of back
7	729	Other disorders of soft tissues
7	244	Acquired hypothyroidism
7	292	Drug-induced mental disorders
7	295	Schizophrenic disorders
7	296	Episodic mood disorders

7	301	Personality disorders
7	304	Drug dependence
7	309	Adjustment reaction
7	338	Pain
7	402	Hypertensive heart disease
7	413	Angina pectoris
7	427	Cardiac dysrhythmias
7	429	Ill-defined descriptions and complications of heart disease
8	569	Other disorders of intestine
9	571	Chronic liver disease and cirrhosis
9	572	Liver abscess and sequelae of chronic liver disease
9	574	Cholelithiasis
9	577	Diseases of pancreas
9	70	Viral hepatitis
9	287	Purpura and other hemorrhagic conditions
9	284	Aplastic anemia and other bone marrow failure syndromes
9	286	Coagulation defects
9	303	Alcohol dependence syndrome
10	573	Other disorders of liver
11	575	Other disorders of gallbladder
12	592	Calculus of kidney and ureter
13	596	Other disorders of bladder
14	614	Inflammatory disease of ovary
15	620	Noninflammatory disorders of ovary
16	110	Dermatophytosis
17	625	Pain and other symptoms associated with female genital organs
18	626	Disorders of menstruation and other abnormal bleeding from female genital tract
19	642	Hypertension complicating pregnancy
20	646	Other complications of pregnancy
21	647	Infectious and parasitic conditions in the mother classifiable elsewhere
22	648	Other current conditions in the mother classifiable elsewhere
23	649	Other conditions or status of the mother complicating pregnancy
24	654	Abnormality of organs and soft tissues of pelvis
25	659	Other indications for care or intervention related to labor and delivery
26	153	Malignant neoplasm of colon
27	681	Cellulitis and abscess of finger and toe
28	197	Secondary malignant neoplasm of respiratory and digestive systems
29	199	Malignant neoplasm without specification of site
30	211	Benign neoplasm of other parts of digestive system
31	730	Osteomyelitis
32	731	Osteitis deformans and osteopathies associated with other disorders classified elsewhere
33	242	Thyrotoxicosis with or without goiter
34	723	Other disorders of cervical region
35	251	Other disorders of pancreatic internal secretion
36	266	Deficiency of B-complex components
37	293	Transient mental disorders due to conditions classified elsewhere
38	298	Other nonorganic psychoses
39	820	Fracture of neck of femur
40	332	”Parkinsons disease
41	333	Other extrapyramidal disease and abnormal movement disorders
42	337	Disorders of the autonomic nervous system
43	346	Migraine

44	355	Mononeuritis of lower limb
45	356	Hereditary and idiopathic peripheral neuropathy
46	365	Glaucoma
47	389	Hearing loss
48	397	Diseases of other endocardial structures
49	218	Uterine leiomyoma
50	424	Other diseases of endocardium
51	441	Aortic aneurysm and dissection
52	444	Arterial embolism and thrombosis
53	447	Other disorders of arteries and arterioles
54	456	Varicose veins of other sites
55	969	Poisoning by psychotropic agents
56	465	Acute upper respiratory infections of multiple or unspecified sites
57	477	Allergic rhinitis
58	490	Bronchitis
59	507	Pneumonitis due to solids and liquids

Table 8. Low Income Community Detection - Walktrap

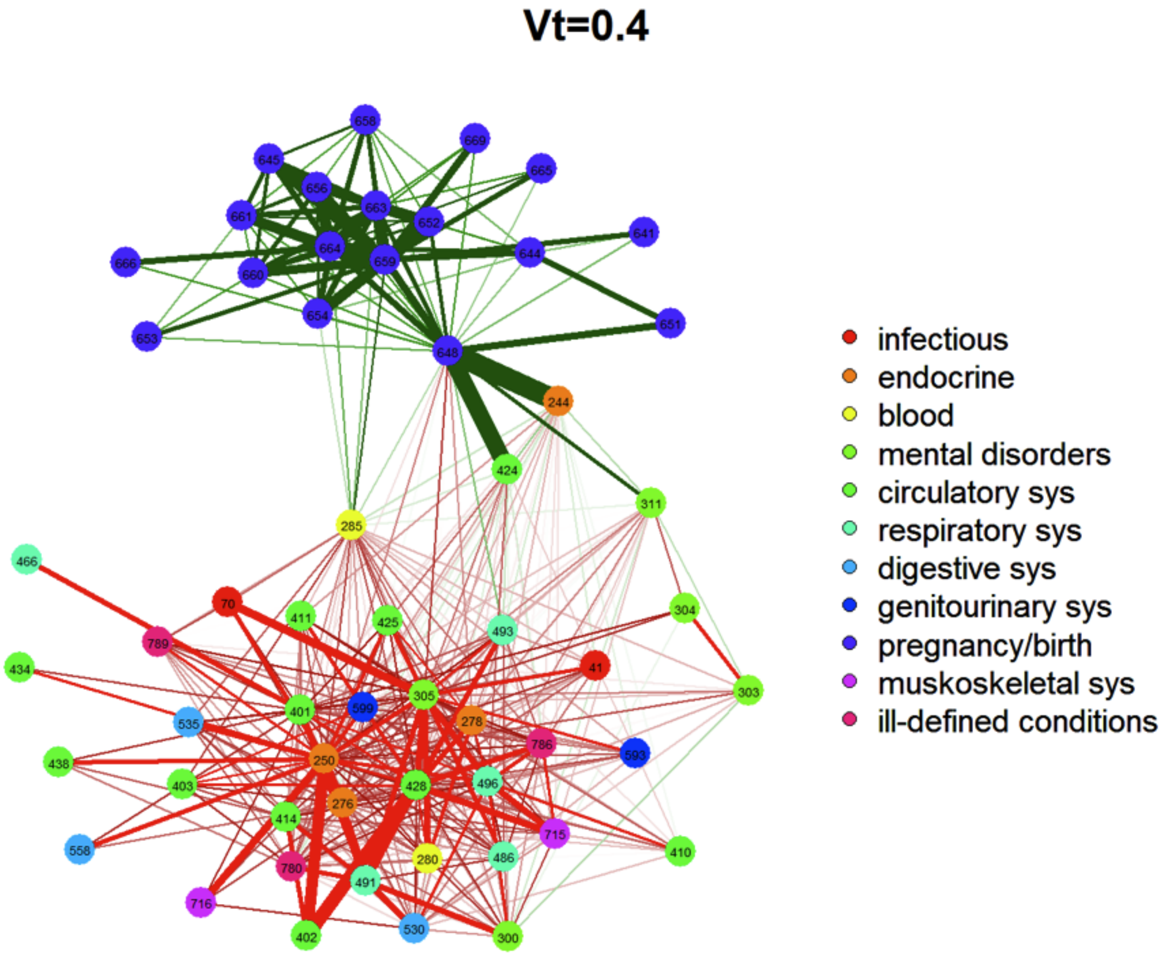

Figure 2. Network representation of over-represented Private Insurance diagnoses compared to Medicaid
